# Radiation Therapy for Secondary Cutaneous Plasmacytomas

**DOI:** 10.1155/2013/739230

**Published:** 2013-10-24

**Authors:** Jerry Liu, Richard Bakst, Robert Phelps, Sundar Jagannath, Seth Blacksburg

**Affiliations:** ^1^Department of Radiation Oncology, Icahn School of Medicine at Mount Sinai, New York, NY 10029, USA; ^2^Department of Pathology, Icahn School of Medicine at Mount Sinai, New York, NY 10029, USA; ^3^Department of Medical Oncology, Icahn School of Medicine at Mount Sinai, New York, NY 10029, USA

## Abstract

We are reporting a case of a 75-year-old man with multiply recurrent IgA-lambda multiple myeloma status post multiple rounds of chemotherapy, autologous stem cell transplantation, and palliative radiation therapy for diffuse bone lesions. Approximately 15 years after original diagnosis, he developed secondary cutaneous plasmacytomas of the right arm, right chest wall, and right upper back over the course of several months. He underwent palliative involved field 3D conformal photon or en face electron therapy concurrently with various chemotherapy regimens and achieved good to complete response with palliation of pain at the irradiated sites. He died of complications related to his disease approximately 7 months after developing skin lesions. The case presented is unique for dermal involvement of myeloma treated with palliative involved field radiation.

## 1. Introduction

Multiple myeloma (MM) is a plasma cell malignancy with clonal proliferation of neoplastic plasma cells in bone marrow with monoclonal immunoglobulin production leading to skeletal destruction. Cutaneous involvement from these tumors is usually in the setting of direct extension from underlying plasmacytomas. Skin plasmacytomas not associated with underlying lesions, also known as secondary cutaneous plasmacytomas (SCP), are rare with less than 100 reported cases [1–17]. Development of these lesions is often a reflection of high systemic tumor burden in the late stages of disease and is a poor prognosticator with most patients dying within 12 months [1, 2].

Few cases are reported in the literature documenting use of radiation therapy to treat SCPs [1, 2, 11, 14]. Appropriate management of these cases, in regards to use of radiation therapy, is not established. This report presents a case of SCPs treated with involved field radiation therapy. 

## 2. A Case Report

 In September 1996, a 58-year-old white man presented with left scapular discomfort and subsequent workup suspicious for plasmacytoma. The original bone marrow biopsy revealed plasmacytosis of 30%, and immunofixation demonstrated high IgA and free lambda light chain. Original laboratory tests revealed 1.2 g/dL M protein with normal albumin, hemoglobin, renal function, calcium, and LDH. He was without Bence-Jones proteinuria. Imaging demonstrated left scapular, right clavicular, sternal, and bilateral humeral lytic bony lesions. He was diagnosed with Durie-Salmon Stage IIIA, International staging I, and IgA-lambda MM. 

He underwent 3 cycles of bortezomib, doxorubicin, and dexamethasone (VAD) chemotherapy with excellent response. Labs and bone marrow biopsy in January 1997 demonstrated complete remission, and he completed cyclophosphamide therapy with stem cell harvest. This was followed by his first autologous stem cell transplantation (SCT) in March 1997 with a rapid and complete hematopoietic recovery. He then underwent melphalan therapy followed by his second autologous SCT in August 1997 with a rapid and complete hematopoietic recovery. This was followed by involved field external beam radiation therapy (EBRT) to the left scapula (4140 cGy in 23 fractions) completed in January 1998. He was started on pamidronate in May 1998 and underwent idiotype vaccination in 1999.

He remained in remission until February 2005 when he was found on surveillance to have elevated IgA and free lambda light chains. Imaging workup was unrevealing of new bony disease. Bone marrow biopsy demonstrated 15% plasmocytosis with 45% cellularity. He was started on thalidomide and dexamethasone therapy in May 2005 with good response. He was then placed on maintenance thalidomide, dexamethasone, and cyclophosphamide in October 2006. He was found to have near complete response, and maintenance chemotherapy was stopped in April 2007. He was found in August 2008 to be in complete remission. 

He remained in complete remission until July 2011 when he presented in followup with right thigh pain associated with MRI findings of multiple lumbar vertebral lesions and right femoral shaft lesion with significant cortical erosion. He underwent open resection of the tumor and intramedullary rod fixation of his right femur with pathology consistent with plasma cell tumor. He subsequently underwent 1 cycle of bortezomib, cyclophosphamide, and dexamethasone (VCD) and 4 cycles of bortezomib, lenalidomide, and dexamethasone (VRD) with complete remission achieved in October 2011. Subsequently, he was maintained on lenalidomide until June 2012 and was found on positron emission tomography and computed tomography (PET-CT) to have multiple new hypermetabolic lesions diffusely. He then underwent 2 cycles of VRD followed by 2 cycles of carfilzomib, cyclophosphamide, and dexamethasone. During this time he completed a course of palliative EBRT to the proximal left clavicle (3000 cGy in 15 fractions) in September 2012 with resolution of pain and decrease in swelling at that site. 

During the prior treatment, he was found to have acute onset right arm swelling soon after right-sided chest wall Port-A-Cath placement. MRI performed shortly afterwards demonstrated a large humeral lesion associated with a soft tissue component. His chemotherapy was changed to dexamethasone, cyclophosphamide, etoposide, and cisplatin (DCEP) with plans for repeat autologous SCT. He underwent tumor curettage and intramedullary nailing of the right humerus in October 2012 and subsequently completed a course of palliative EBRT (3000 cGy in 10 fractions) to the entire right humerus with significant improvement in right arm pain and swelling. 

**Figure 1:**
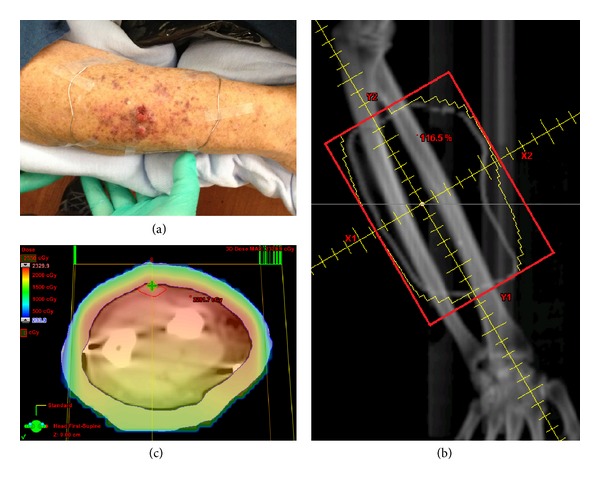
Secondary cutaneous plasmacytoma presenting as (a) nodularity on patchy violaceous background at dorsal surface of patient's right forearm. (b) Right forearm AP treatment field, as outlined in red, with (c) dosimetry shown on axial planning computed tomography image.

**Figure 2:**
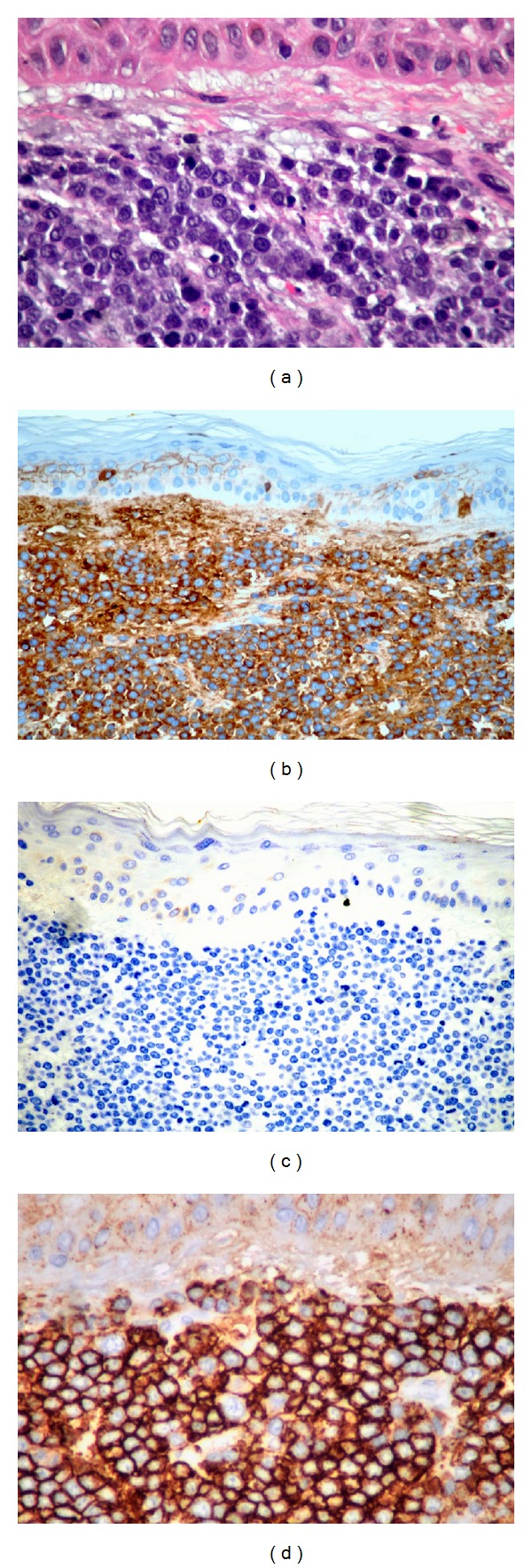
Histopathologic features of biopsy from patient's right forearm lesion demonstrating: (a) neoplastic plasma cells infiltrating dermis with pleomorphic nuclei on Haematoxylin-Eosin staining, (b) strong lambda light chain staining, (c) lack of kappa light chain expression, and (d) strong staining for CD138 (plasma cell marker).

**Figure 3:**
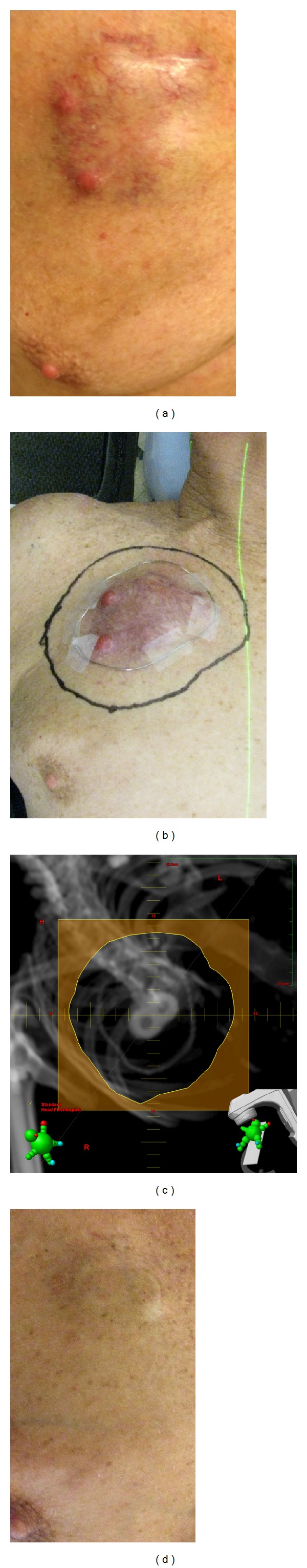
Secondary cutaneous plasmacytoma presenting as (a) nodularity on violaceous background overlying Port-A-Cath on patient's anterior right chest wall. (b) Treatment positioning with outlined target and (c) en face field with custom block on simulation image. (d) Full resolution of lesions observed one month after treatment.

Bone marrow biopsy in November 2012 was without evidence of disease, but PET-CT findings demonstrated new hypermetabolic lesions in the left humerus, pelvis, and femur associated with intramedullary soft tissue densities. He also developed multiple cherry red nodules on a patchy violaceous background at the dorsal surface of his right forearm (Figure 1(a)). Punch biopsy of one of these lesions revealed monomorphous proliferation of atypical lymphoid cells with eccentric clock face nuclei. Immunohistochemistry was positive for lambda positive, kappa negative, CD138 positive staining (Figures 2(b)–2(d)), and CD3 and CD20 staining. This was consistent with his initial diagnosis of myeloma and represented his first presentation of SCPs. He also concurrently developed red circular nodules on a violaceous background overlying his Port-A-Cath site on his anterior right chest wall (Figure 3(a)) which prevented its use for chemotherapy administration. He was started on bortezomib and bendamustine therapy.

The patient then completed a course of palliative EBRT (3000 cGy in 10 fractions via AP field with 1 cm bolus prescribed to 3.5 cm depth to account for contour of arm) to the entire violaceous area of his right forearm (Figures 1(b) and 1(c)) in December 2012. This was followed by an electron boost (200 cGy in 1 fraction of planned 1000 cGy in 5 fractions via en face 6 MeV beam with 0.5 cm bolus) which was discontinued by the patient due to irritation from dry desquamation and erythema. At the end of treatment, the violaceous background had faded, and the nodules were slightly smaller in size with resolution of pain. He also underwent a course of electron beam therapy (3000 cGy in 10 fractions via en face 12 MeV with 0.5 cm bolus) (Figures 3(b) and 3(c)) to the right chest wall resulting in full resolution of the lesions at this area, allowing use of his Port-A-Cath for chemotherapy (Figure 3(d)). 

During this time, he also presented with acute onset right sided facial weakness and slurred speech associated with MRI findings of a 2.2 cm neoplastic deposit at the right skull base involving the right occipital bone, intimately associated with adjacent jugular bulb with adjacent abnormal enhancement of the right facial nerve. He underwent a course of palliative EBRT (3250 cGy in 13 fractions) to the right base of skull lesion resulting in stabilization of his partial right facial palsy. 

By January 2013, the patient was experiencing progressive right arm lymphedema severely limiting use of the extremity. PET-CT demonstrated diffuse progression of his disease with new hypermetabolic visceral, musculoskeletal, and chest wall lesions. He also developed new subcutaneous lesions of the bilateral breasts, right leg, and left arm. Biopsies of new subcutaneous lesions of the right breast and right shin revealed pathologic findings consistent with SCPs. In February 2013, he completed 2 cycles of low-dose carmustine and melphalan therapy with autologous stem cell boost. He experienced mixed response with resolution of his chest wall SCPs, though his right arm was progressively more lymphedematous, and new skin lesions were appearing throughout both arms. He was started on pomalidomide, carfilzomib, and dexamethasone in March 2013. 

After long discussion regarding risks of progressive lymphedema and compartment syndrome with further radiation of his right upper arm, the patient remained adamant about proceeding with local treatment. He completed a repeat course of palliative EBRT (3000 cGy in 15 fractions with 1 cm bolus) to his right arm from shoulder to wrist in April 2013. He experienced moderate response of the right arm SCPs during the course of treatment and, with use of compression sleeve, slight improvement in right arm edema. There was, however, progression of skin lesions superior and proximal to the treatment field and to upper right back. He underwent electron beam therapy (1500 cGy of planned 3000 cGy in 5 of 10 planned fractions via en face 9 MeV with 0.5 cm bolus) to his upper back with some visual improvement of dermal lesions at the treated area and was also planned to undergo a course of conformal EBRT to the right shoulder. His radiation and systemic therapy were discontinued in mid-April 2013, however, due to progressive worsening renal function associated with diffuse visceral, musculoskeletal, and dermal progression of disease. He was arranged for home hospice and died of renal failure in early May 2013. 

## 3. Discussion

Plasma cell malignancies include classic MM, extramedullary primary plasmacytoma, solitary plasmacytoma of the bone, and plasma cell leukemia. Each of these shares the pathophysiology of malignant clonal proliferation of plasma cells with monoclonal immunoglobulin production. In MM, monoclonal proteins are detected in the blood or urine, at least 10% clonal plasma cells are present in bone marrow or plasmacytomas, and end-organ damage is demonstrated (anemia, bony destruction, hypercalcemia, or renal failure) [18]. Plasmacytomas, in the setting of MM, can reside in the bone and/or soft tissue (secondary extramedullary plasmacytoma). Cutaneous involvement is uncommon, occurring in approximately 2% of cases [19], and is usually in the setting of direct extension from underlying bone or soft tissue lesions. More rarely, dermal metastases, SCPs, can develop from lymphatic or hematologic extension, usually in the setting of late stage disease with high tumor burden [1–3]. 

First reported by Bloch in 1910 [20], the characteristics of SCPs have been well described in the largest review series by Patterson et al. [1] and Raquena et al. [2, 3]. SCPs have consistently been described as erythematous or violaceous nodules and plaques with a varied anatomical distribution and most commonly present on the trunk and abdomen and less commonly on the extremities or head and neck region. Multiple nodules are almost always present. Ulceration of lesions with secondary infections has also been described. There may also be a predilection of sites of previous or current inflammation, which has been described in the setting of leukemia cutis [21–23].

 On histopathology, SCPs have been shown in either nodular or interstitial patterns. Nodular pattern is more frequently seen and demonstrates malignant plasma cell clusters arranged in cohesive masses. Interstitial pattern consists of narrow cords of tumor cells dispersed between connective tissue bundles. In both patterns, malignant plasma cells show varying stages of atypia and contain monoclonal immunoglobulins matching that of the originally diagnosed MM [2]. The preponderance of IgG MM in reported cases of SCPs seems to reflect the overall pattern of monoclonal immunoglobulin frequency in all MM patients [1–3, 17]. Reported literature does show, however, a disproportionately high frequency of IgD MM in patients with SCP as well as more aggressiveness of disease in these cases [1, 2, 24]. Staining of the SCP tumor cells for immunomarkers usually reveals positivity for CD79a, CD138 (syndecan-1, plasma cell specific marker), and epithelial membrane antigen (EMA), as well as negativity for pan-B-cell antigens, CD19, and CD20 [2]. 

 Management of SCPs in reported cases has mostly consisted of combination chemotherapies to address rising systemic burden of disease. The use of radiation therapy to address cutaneous plasmacytomas, primary or secondary, has been reported in a few cases, with good response and/or palliation [1, 2, 14, 25–27]. Radiation techniques and clinical response to treatment have not, however, been well described. Only a single report by Nguyen and Dagnault [14] provided information regarding the radiation regimen. A conformal 5-field electron therapy plan (6 MeV with 0.5 cm bolus) to 2000 cGy in 5 fractions was used to treat SCPs of the right lower leg. Their patient achieved near complete response at 1-month followup but later developed SCPs diffusely outside of the treated area and underwent additional EBRT to those sites. The patient ultimately died of pneumonia as complication of chemotherapy approximately 7 months after initial presentation of SCPs. 

Consistent with reported literature, the development of SCPs in our patient was coincident with increasing systemic tumor burden and portended a poor prognosis (7-month survival after presentation). Biopsies demonstrated that his lesions were histopathologically consistent with his diagnosed MM with expected immunomarker profile. Our patient underwent limited field photon or electron EBRT to total doses of 2000–3000 cGy in 200 cGy fractions that achieved successful palliation with good-complete response of SCPs.

In conclusion, we believe that this case is an important contribution as there is a scarcity of the literature regarding the use of EBRT in addressing SCPs in the setting of MM. Though the presence of these lesions portends a poor prognosis, palliation of pain and local control of the involved areas remain worthy goals for treatment. We demonstrate that palliative EBRT can achieve these goals while being well tolerated. Further reporting of similar cases is important to help determine optimal management.
